# Experimental evolution reveals that high relatedness protects multicellular cooperation from cheaters

**DOI:** 10.1038/ncomms11435

**Published:** 2016-05-03

**Authors:** Eric Bastiaans, Alfons J. M. Debets, Duur K. Aanen

**Affiliations:** 1 Department of Plant Sciences, Laboratory of Genetics, Wageningen University, Wageningen 6708 PB, The Netherlands

## Abstract

In multicellular organisms, there is a potential risk that cheating mutants gain access to the germline. Development from a single-celled zygote resets relatedness among cells to its maximum value each generation, which should accomplish segregation of cheating mutants from non-cheaters and thereby protect multicellular cooperation. Here we provide the crucial direct comparison between high- and low-relatedness conditions to test this hypothesis. We allow two variants of the fungus *Neurospora crassa* to evolve, one with and one without the ability to form chimeras with other individuals, thus generating two relatedness levels. While multicellular cooperation remains high in the high-relatedness lines, it significantly decreases in all replicate low-relatedness lines, resulting in an average threefold decrease in spore yield. This reduction is caused by cheating mutants with reduced investment in somatic functions, but increased competitive success when fusing with non-cheaters. Our experiments demonstrate that high genetic relatedness is crucial to sustain multicellular cooperation.

## Abstract (web summary)

Maintenance of cooperation in multicellular organisms is hypothesized to depend on high relatedness among cells. Here, Bastiaans *et al*. provide empirical support for this hypothesis by directly comparing the evolutionary stability of multicellular cooperation in experimental lines of a fungus kept at either high or low relatedness.

## Graphical abstract

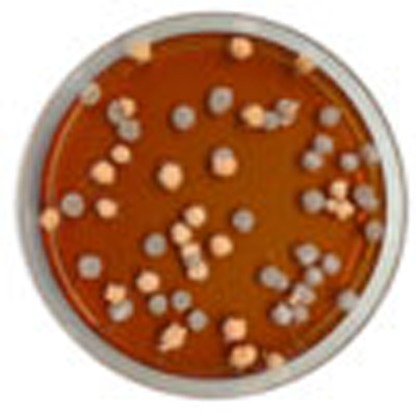

The origin of multicellularity was one of the major transitions in evolution^1^,^2^,^3^. It allowed increases in size and complexity through division of labour between differentiated cellular lineages within an individual^4^. The most significant division of labour from an evolutionary perspective was in reproduction. Only a fraction of the cells reproduce, whereas the majority altruistically support the reproductive cells by contributing to somatic functions. Such reproductive division of labour leads to a potential conflict among the cells of multicellular individuals. Cheating variants with an increased probability to become a reproductive cell will be selected within the individual, even if such variants decrease individual fitness^5^. The stability of multicellular growth thus depends on mechanisms to reduce the selective scope for cheats.

High-genetic relatedness has been proposed as the fundamental factor contributing to the stability of multicellular growth and of other major transitions in evolution^2^,^3^,^6^,^7^. In the vast majority of multicellular organisms, individuals develop from a single-celled zygote undergoing mitotic divisions. The resulting high-genetic relatedness (coefficient of relatedness, *r*=1, barring new mutations) implies that some of these cells can become sterile somatic tissues in the service of other cells if this increases the total reproduction of the group of cells (that is, if benefits>costs)^3^,^8^.

Although there is circumstantial and comparative phylogenetic support for the hypothesis that high relatedness stabilizes multicellular growth^6^, and experimental evidence that low-relatedness conditions can lead to the collapse of multicellular cooperation^9^,^10^, direct tests of this hypothesis are rare and suboptimal. Organisms that arise via aggregation of solitary cells instead of clonal outgrowth from a single-celled zygote provide an opportunity to experimentally reduce among-cell relatedness. The best-known examples are cellular slime molds (*Dictyostelium* spp.) and myxobacteria (*Myxococcus* spp.), which form multicellular fruiting bodies from aggregating single cells. It has been shown that cheaters can arise under low-relatedness conditions^9^,^10^. However, direct comparisons between high- and low-relatedness conditions under otherwise identical conditions have so far not been made, since relatedness cannot easily be controlled during evolution experiments^11^.

Here, we use the fungus *Neurospora crassa* as a model system to test the significance of genetic relatedness for the stability of multicellular growth. Fungi are intermediate between pure clonal development from a single-celled zygote and development via aggregation, as they can increase in size by mitotic division, but also by fusion between the filaments of different individuals^12^,^13^; this characteristic allowed us to vary relatedness. Filamentous fungi form filaments (hyphae) that branch and fuse regularly to form a dense, radially growing network called a mycelium, or fungal colony, and each fragment can reproduce via the formation of sexual and asexual spores. In contrast to most other multicellular organisms, cell compartmentalization is not very strong and in *N. crassa* nuclei can freely move through parts of the mycelium^14^. Therefore, the cooperating units in the fungal colony primarily are the haploid nuclei^12^.

Using experimental evolution under low- and high-relatedness conditions, we find that multicellular cooperation in this fungus remains high in the high-relatedness lines, while it significantly decreases in all replicate low-relatedness lines, resulting in an average threefold decrease in spore yield. This reduction is caused by cheating mutants with reduced investment in somatic functions, but increased competitive success when fusing with non-cheaters. These experiments demonstrate that high-genetic relatedness is crucial to sustain multicellular cooperation.

## Results

### Varying relatedness during experimental evolution

**Figure 1:**
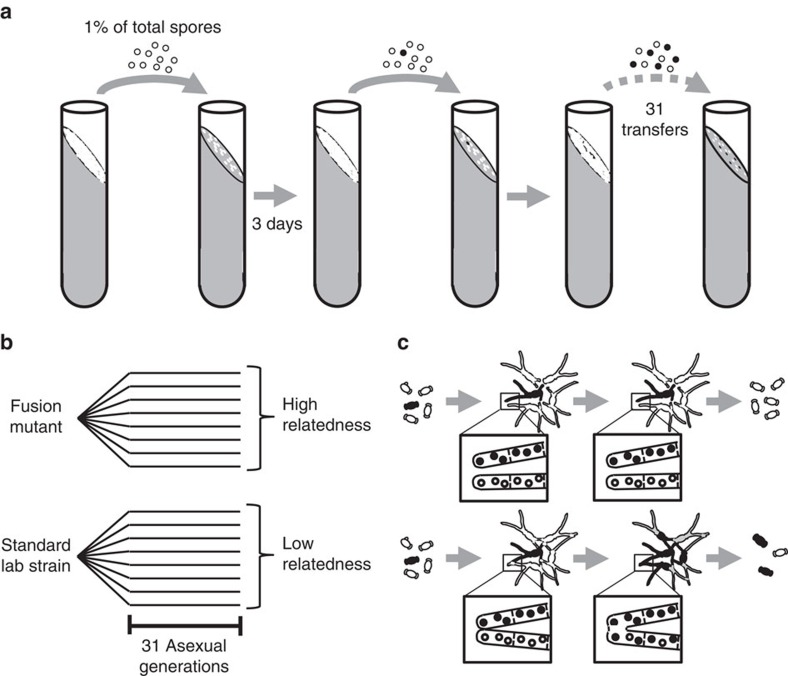
Experimental setup and the consequences of relatedness for cheating. (**a**) Experimental setup. For each evolution line, asexual spores were inoculated on agar and after 3 days growth all newly formed asexual spores were collected (∼1 × 10^8^ spores), of which 1% were inoculated on a fresh tube. This cycle was repeated for 31 generations. (**b**) Eight replicate evolution lines were started from a single asexual spore of the standard lab strain (low-relatedness treatment), and eight lines from a spore of the fusion mutant (high-relatedness treatment). (**c**) Fusion between germinating spores results in mixing of cytoplasm and nuclei, thereby lowering relatedness among the nuclei within a colony (lower panel). Low relatedness facilitates selection at the level of the nuclei within the colony, which may favour variants with an increased probability to reproduce, even if they negatively impact total spore production. Such cheater morphotypes owe their increased fitness to fusion with non-cheating genotypes. If fusion is not possible, the nuclei from different individuals remain separated, thereby maintaining high relatedness among nuclei within an individual (upper panel). High relatedness implies that selection primarily acts among different individuals, disfavouring cheating mutants, as each genotype will be held accountable for its contribution to somatic functions required to produce the optimal number of spores.

To experimentally test the importance of high relatedness among nuclei for selection against cheating variants, we performed an evolution experiment by serial culturing via transfer of asexual spores. We used two treatments, one maximizing and the other minimizing relatedness among the nuclei of individuals (Fig. 1a–c). In the low-relatedness treatment, we used the standard lab strain, which can freely fuse, in an environment of high-inoculation density and complete mixing at each transfer. The use of a single allotype and a high-inoculation density guaranteed fusion among all germinated spores, so that a single, coherent fungal colony was formed after each transfer. Essentially, this made the whole culture a common good. We predicted that these conditions would favour cheating, as the link between investment in somatic ‘helper' tissue and reproduction was minimized (Fig. 1c). The other treatment was identical except that, to maintain maximal relatedness, we used a mutant that lacked the ability to fuse. In this treatment, each germinating spore forms a separate individual, which produces offspring. This maximizes the relatedness among the nuclei of a single individual, and minimizes the opportunities for cheating, as a cheating nucleus arising within an individual will be tested for its effect on multicellular fitness in the next round of experimental evolution (that is, it is selected on the basis of its contribution to somatic growth as well; Fig. 1c).

### Low relatedness selects for reduced yield and slower growth

**Figure 2:**
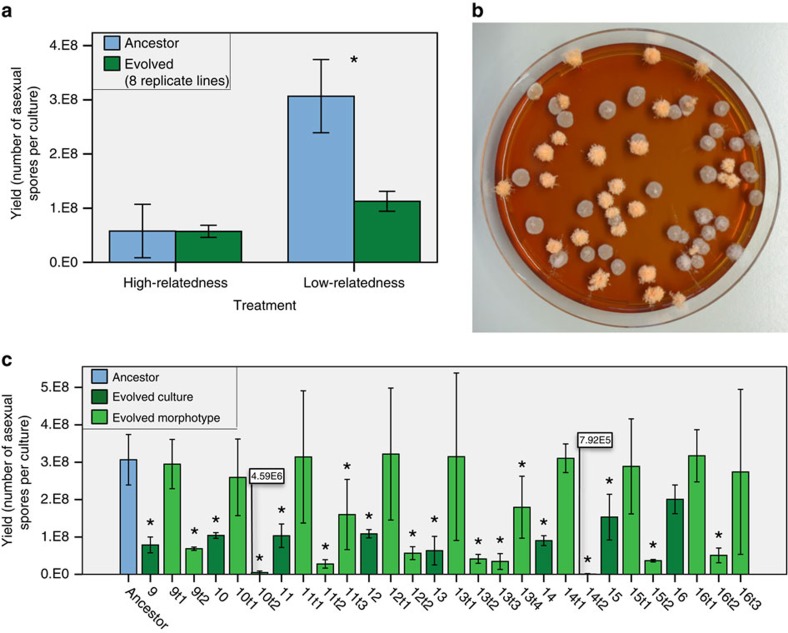
Yield of the evolved lines and of morphotypes found within these lines. (**a**) Under high-relatedness conditions high spore production is maintained, while under low-relatedness conditions spore yield drops. Column chart comparing the average yield of eight evolved lines per treatment before and after an estimated 205 asexual generations. Significant changes in yield during evolution (Mann–Whitney's *U*-test; *P<0.05) are indicated. Error bars depict 95% confidence intervals (*n*=3 for ancestor, *n*=24 for evolved). (**b**) Evolution under low-relatedness conditions results in the selection of different morphotypes within all eight lines. Typical photo of a Petri dish with 1-week-old colonies grown from asexual spores of a culture of line 13 evolved for 31 transfers under low-relatedness conditions. Bright-orange colonies are sporulating, similar to the ancestral phenotype, while the pale colonies have reduced sporulation. (**c**) Yield of evolved low-relatedness lines and of the different morphotypes within lines. Column chart comparing asexual spore yields of the low-relatedness lines and of their morphotypes and the ancestral genotype. The first number of labels on the *x*-axis refers to the evolved cultures after 31 transfers (9–16) and the second number to the morphotypes within these lines (9t1–16t3; and to the ancestral culture). Significant differences with the ancestor (Tukey's *post hoc* test *P<0.05) are indicated. Error bars depict 95% confidence intervals (*n*=3).

After 31 rounds of asexual reproduction in 2 × 8 parallel evolving cultures (evolution lines), we determined the average spore yield of all cultures and compared it with the unevolved strain that was used to start the lines (ancestor). In the high-relatedness treatment, spore production had not significantly changed (independent samples Mann–Whitney's *U*-test, *P*=1.000). However, in all replicate lines of the low-relatedness treatment spore production had significantly decreased, on average threefold compared with the ancestor (independent samples Mann–Whitney's *U*-test, *P*=0.001; Fig. 2a). To further investigate the changes in the evolution lines, we assessed variation among single-spore cultures of each line. All low-relatedness lines consisted of at least two morphologically different types (morphotypes; Fig. 2b). Interestingly, some of the high-relatedness lines also consisted of multiple morphologically different types.

**Figure 3:**
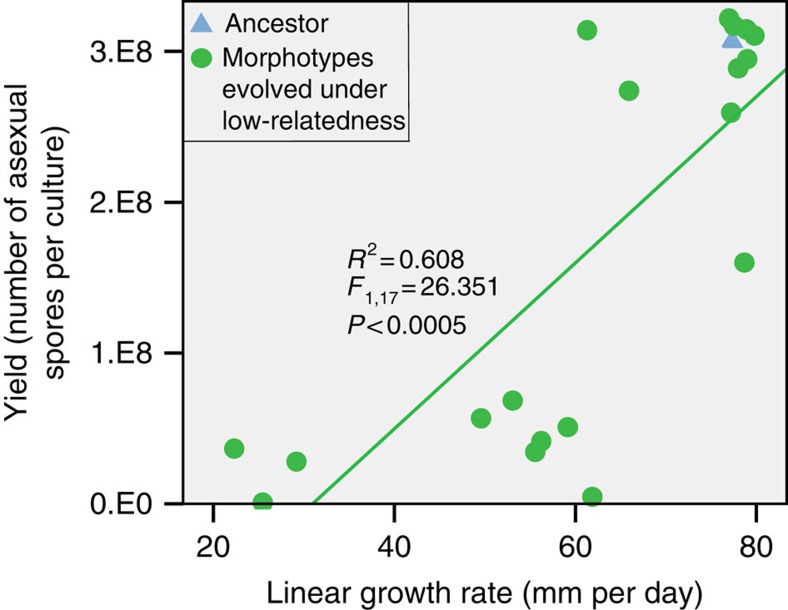
Spore yield and linear growth rate. Comparison between average asexual spore yield (*n*=3) and average linear growth rate of the morphotypes (*n*=3) isolated from the lines evolved under low-relatedness conditions. A significant positive relationship was found between yield and linear growth rate for these types (linear regression, *F*_1,18_=29.62, *P*<0.0005).

To characterize these different types, we isolated the different morphotypes present in low-relatedness lines. We did this by spreading spore suspensions on culture plates and selecting one of each morphologically distinguishable colonies out of more than hundred colonies. We then tested them separately for two fitness components in monoculture, the production of asexual spores (yield), and the mycelial growth rate^15^. Each evolved culture contained at least one morphotype with significantly lower spore yield than the ancestor (Tukey's *post hoc* test *P*<0.05), and at least one type with a spore yield similar to the ancestor (Fig. 2c). Mycelial growth rate was positively correlated with spore yield (linear regression, *F*_1,18_=29.62, *P*<0.0005; Fig. 3). This finding is consistent with the hypothesis that investment in somatic growth results in a high yield later on.

### All low-relatedness lines contain cheater morphotypes

**Figure 4:**
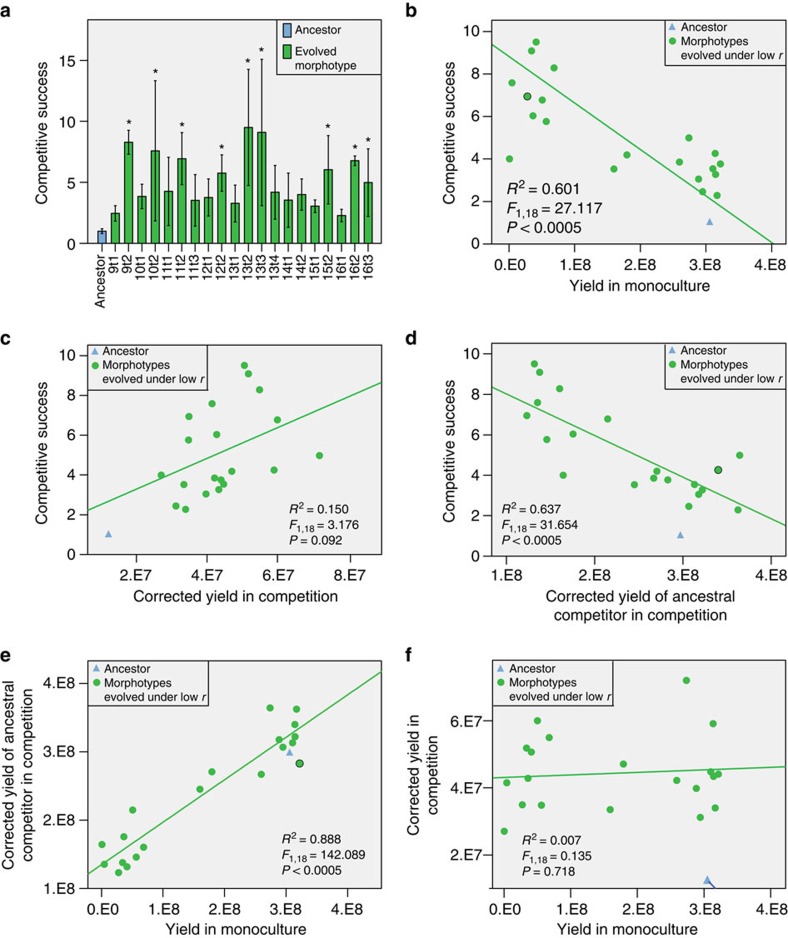
Aspects of competitive success of morphotypes found within evolution lines. Tests of statistical significance of linear regression are in the graphs. (**a**) Competitive success of evolved morphotypes has increased relative to the ancestor. Column chart comparing normalized competitive success of the morphotypes evolved under low relatedness relative to the ancestral strain. Significant differences with the ancestor (Tukey's *post hoc* test *P<0.05) are indicated. Error bars depict 95% confidence intervals (*n*=3). (**b**) There is a highly significant negative correlation between yield in monoculture and competitive success. The graph shows average asexual spore yield (*n*=3) in monoculture plotted against average competitive success (*n*=3) relative to the ancestral strain. (**c**) Competitive success only marginally depends on yield of the evolved type in competition. The graph shows the relation between the averages (*n*=3) of competitive success and corrected yield of the evolved type in competition. The yield is corrected for small deviations in the inoculation frequency from the intended 0.1. (**d**) Competitive success is significantly negatively correlated with the yield of the ancestral type in competition. The graph shows the relation between the averages (*n*=3) of competitive success and the corrected yield of the ancestral type it is competing with. The yield is corrected for small deviations in the inoculation frequency from the intended 0.9. (**e**) There is a highly significant positive correlation between yield in monoculture and the yield of the ancestral competitor in the competition. The graph shows the relation between the average (*n*=3) of corrected yield of the evolved type in monoculture and the average of the corrected yield of the ancestral competitor it is competing with. Corrected yield is corrected for deviations in the inoculation frequency from the intended 0.9. (**f**) There is no significant correlation between the yield of the morphotypes in monoculture and their yield in competition. The graph shows the relation between the average (*n*=3) of corrected yield of the evolved type in competition and its yield in monoculture. Corrected yield is corrected for deviations in the inoculation frequency from the intended 0.1.

We then tested the competitive success of the different morphotypes in competition with their ancestor as a measure of competitive fitness at a low starting frequency relative to the ancestor (1:9; Fig. 4a). We defined competitive success as the ratio between evolved and ancestral spores after one round of competition, relative to that ratio at inoculation (0.1). With three exceptions, the morphotypes with a significant reduction in spore yield in monoculture had a significant increase in competitive success (Tukey's *post hoc* test *P*<0.05), while the other morphotypes did not. This result implies that the genotypes with reduced spore yield in monoculture are obligate cheaters: they invest less in somatic functions, which affects growth and spore production in monoculture, but on access to the ancestral soma they exploit this to obtain higher competitive success. Overall, we found a highly significant negative relationship between competitive success and yield in monoculture (linear regression; *F*_1,18_=27.117, *R*^2^=0.601, *P*<0.0005; Fig. 4b). This tradeoff is typical for obligate cheaters on a social trait, which cannot switch back to producing the common good, in this case the soma, when social morphotypes to cheat on are absent^16^. Of note, within evolved cultures we distinguish two types: the ‘social morphotype' that produces the same number of spores in monoculture as the ancestor, and the ‘cheater morphotype' that produces significantly fewer spores in monoculture.

To confirm that the phenotype of the cheater morphotypes from the low-relatedness lines is caused by nuclear mutations, we back-crossed typical cheater morphotypes to the ancestral type. In all crosses between evolved cheat and ancestral genotypes, progeny segregated 1:1 into the cheating phenotype and the ancestral phenotype, as expected for single-nuclear mutations. In addition, these crosses revealed that cheater morphotypes all have severely reduced female, but not male, functioning, consistent with the hypothesis that these strains have reduced somatic investment required to support the growth of relatively costly female-fruiting structures.

### Success of cheaters depends on reducing yield of competitors

We found a positive but not significant correlation between relative competitive success and absolute spore yield in competition (linear regression, *F*_1,18_=3.176, *P*=0.092; Fig. 4c), showing that competitive success depends only to some degree on absolute spore production in competition. However, we found a highly significant negative correlation between the yield of the ancestral competitor of the evolved morphotype and competitive success of the evolved morphotype (linear regression, *F*_1,18_=31.654, *P*<0.0005; Fig. 4d). These correlations mean that the competitive success of evolved types relative to the ancestor only marginally depends on increased spore production in competition, but very strongly on reduction in yield of the competitor. This finding implies that the social morphotypes pay a high cost in competition with a cheater morphotype, which is in line with the idea that cheaters take resources from the cooperative competitor. This interpretation is further corroborated by the finding that yield in monoculture (the indicator of cheating) is significantly positively correlated with the yield of the ancestor in competition (linear regression *F*_1,18_=142.089; *P*<0.0005; Fig. 4e) and not with the absolute yield of the evolved morphotype in competition (linear regression *F*_1,18_=0.135; *P*=0.718; Fig. 4f).

### Cheating depends largely but not solely on somatic fusion

**Figure 5:**
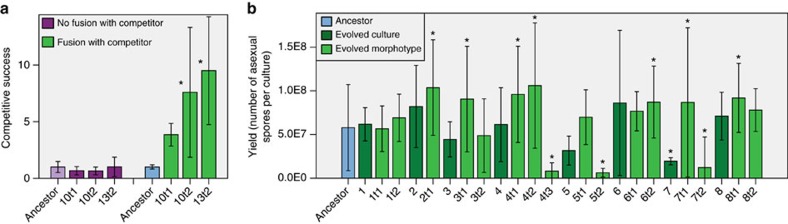
Consequences of restriction to somatic fusion on cheating. (**a**) Increased competitive success depends on somatic fusion. The column chart shows normalized competitive success of three selected morphotypes evolved under low-relatedness conditions relative to an ancestral strain with a different allotype with which fusion is not possible. For comparison, competitive success of the same morphotypes relative to the ancestral strain with which fusion is possible is depicted in the right-hand graph. Significant differences with the ancestor (Tukey's *post hoc* test *P<0.05) are indicated. Error bars depict 95% confidence intervals (*n*=3). (**b**) Column chart comparing asexual spore yields of the high-relatedness lines and the morphotypes within these lines with the yield of the ancestral type. No significant differences with the ancestor were found using a Tukey's *post hoc* test; significant differences with the ancestor using the less conservative LSD *post hoc* test (*P<0.05) are indicated. Error bars depict 95% confidence intervals (*n*=3).

We hypothesized that the selection of cheater morphotypes in the low-relatedness treatment depended on fusion between individuals, which is not possible in the high-relatedness treatment, and is not due to potential other differences between the strains used. To test this hypothesis, we determined the competitive success of three evolved morphotypes relative to a near-isogenic ancestor they could not fuse with. We found no significant difference in competitive success of the cheater morphotypes compared with the ancestor when fusion was blocked (Tukey's *post hoc* test, *P*>0.05; Fig. 5a). This result confirms that fusion is an important determinant of the competitive success of the cheater morphotypes in competition with the (social) ancestral type. However, if the success of the cheater morphotype in competition with the ancestral type solely depended on fusion, a competitive disadvantage would be expected in the absence of fusion, since these cheater morphotypes have highly reduced spore production in monoculture compared with the ancestor (∼64- and ∼7-fold reductions for 10t2 and 13t2, respectively).

The incongruence between spore production in monoculture and relative spore production in competition with an incompatible strain is most likely due to alternative possibilities to cheat in the absence of successful fusion. One possibility is that the evolved types profit from the cytoplasmic contents of their incompatible competitors on cell death due to incompatibility, albeit to a lesser extent than with successful fusion. An important detail of vegetative incompatibility in ascomycetes is that incompatibility occurs upon hyphal fusion. So: hyphal fusion is the default, and then either cell death occurs (if a combination is incompatible) or an integrated larger individual is formed (if a combination is compatible). Therefore, in incompatible interactions a fraction of the cells die, and the cytoplasmic content of these cells may become available to the surviving cells. A related possibility is that cheating occurs on leaked or excreted goods such as extracellular enzymes that can be accessed by the evolved type, but again to a lesser extent than with successful fusion. The latter explanation is consistent with the finding that some of the high-relatedness lines also contain some morphotypes with reduced spore yield in monoculture (Fig. 5b). Although we did not measure competitive success of these types, this result again suggests that there are additional possibilities to cheat on neighbouring mycelia in the absence of fusion. Irrespectively of the exact reason for the apparent incongruence between the single-strain spore yields and the competitive success data, this experiment demonstrates that competitive success is highly reduced in competition with an incompatible strain, compared with competitive success in competition with a compatible strain. This result shows that the majority of increased competitive fitness is due to successful fusion.

### Negative-frequency dependence can lead to stable coexistence

**Figure 6:**
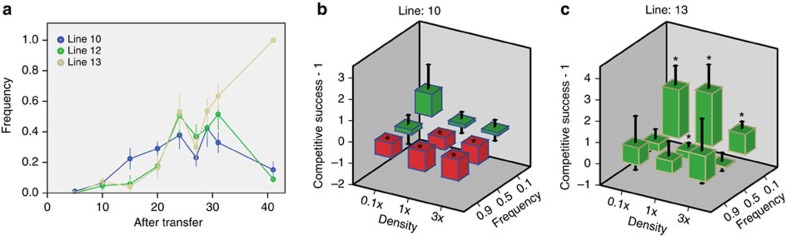
Frequency and density dependence of competitive success of cheaters. (**a**) Line chart showing the frequency changes of the cheater morphotypes during the evolution experiment in three lines. (**b**,**c**) Column diagrams showing competitive success of the cheater morphotypes relative to the social morphotypes from line 10 and 13 as a function of inoculation density (*x*-axis) and inoculation frequency (*z*-axis). On the *y*-axis competitive success—1 is depicted, so that positive (green bars) and negative values (red bars) represent a selective advantage or disadvantage to the competitor, respectively. Competitive success is measured for different inoculation densities (*x*-axis) and different starting frequencies (*z*-axis). Starting density 1 × is the density representative for the first few transfers of the evolution experiment (4 × 10^7^ spores per ml). The other starting densities are three times concentrated (3 × ) and 10 times diluted (0.1 × ) relative to 1 × . Significant differences from 0 (*t*-test *P<0.05) are indicated. Error bars depict 95% confidence intervals (*n*=3).

After 31 transfers in all the evolved cultures from the low-relatedness treatment, a social morphotype still coexisted with one or more cheating morphotypes. To test if this polymorphism was stable, or if the cheating type would go to fixation, we followed the dynamics of three lines during the evolution experiment (Fig. 6a) and continued the evolution experiment for all low-relatedness lines for another 10 transfers (Table 1). Except for one line (line 13), all lines contained both the cheating and the social morphotype after 41 transfers. Stable coexistence of the different types could formally be explained if the interaction between types were mutually beneficial. We can reject this hypothesis, since the yield of all evolution lines was intermediate between the yields of the composite types in monoculture, and not higher, as predicted under the mutualism model.

Alternatively, negative frequency-dependent selection may explain the stable coexistence of cheater and social morphotypes. Negative frequency-dependent selection has been found in several studies on social cheating in microbes (for example, see ref. 17), and has been theoretically predicted when there is population structuring and an association between the level of cooperation and population growth^18^, both of which conditions are fulfilled in our experiments. To test for negative frequency-dependent selection and the effect of inoculation density, we measured the competitive fitness of two cheater morphotypes with the social morphotype with which they were coexisting after transfer 31 at varying densities and frequencies. The lines from which the cheater morphotypes were selected, lines 10 and 13, showed opposite patterns of population dynamics (Fig. 6a). Indeed, the competition experiments showed a sharp contrast between line 10 and 13, which explains the frequency changes of the cheater morphotypes in these lines (Fig. 6b,c). In line 10, we only measured an advantage for the cheater morphotype at a low frequency, while at the highest frequency there was a significant disadvantage, explaining that the cheater morphotype remained at a low frequency even after prolonged evolution. So for line 10, we confirmed that negative frequency-dependent selection acts on the cheater morphotype. In contrast, in line 13, the cheater morphotype always had an advantage relative to the more social morphotype, which fits the observation that this type went to fixation. We did not observe a consistent effect of density, so we do not know the exact nature of negative-frequency dependence. A non-mutually exclusive hypothesis is that coevolution between the various types of a line has occurred, maintaining the frequency of cheater morphotypes low due to the evolution of resistance. Such coevolution has recently been demonstrated in *Dictyostelium*^19^.

## Discussion

Our results experimentally demonstrate that high genetic relatedness is crucial for the evolutionary stability of multicellular cooperation, as multicellular cooperation breaks down easily due to selection of cheater morphotypes under low-relatedness conditions. In nature, the low-relatedness conditions of our evolution experiments are unlikely to be met, and, hence, the long-term opportunities for cheating variants seem restricted. Firstly, single-celled bottlenecks usually are associated with dispersal (which may have been the initial advantage of alternation between unicellular and multicellular stages^20^,^21^), so that—compared with our experiments—there is more opportunity for clonal outgrowth, selecting against cheater morphotypes. Secondly, highly diverse genetic allorecognition mechanisms in *Neurospora*^22^ restrict somatic fusion to close relatives only. Although fusion provides benefits relative to rejection, due to increased individual size and the absence of costly antagonism^23^, allorecognition has been found in the vast majority of organisms that can fuse, supporting the hypothesis that high relatedness is crucial for the evolutionary stability of multicellular growth^7^,^24^. Although our results demonstrate that somatic fusion provides the most pervasive opportunities for cheating, the finding that evolved cheater morphotypes in competition with an incompatible ancestor do not show the full disadvantage they have in monoculture suggests that leaked or excreted cytoplasmic contents also are prone to cheating in this fungus. Preventing successful fusion due to allorecognition thus does not exclude all possibilities to cheat.

Control of conflicts is essential not only for the stability of multicellular cooperation, but also for the stability of other major transitions in evolution, such as the transition from solitary individuals to highly integrated societies of social insects^2^. Our study complements other experimental studies demonstrating the importance of high relatedness for the stability of cooperation at other hierarchical levels of biological organization, such as between bacteria cooperating in excreted common goods^25^ and eusocial insects cooperating in reproduction^26^.

Additional mechanisms may contribute to the maintenance of social groups, once they are formed^27^. For example, in colonies of social insects policing mechanisms exist to prevent worker reproduction^28^. Analogously, in higher organisms, such as mammals, policing mechanisms reduce the selective scope for cancerous mutations^29^. Furthermore, in most animals, an early germline sequestration during development limits the selective opportunities of selfish mutants^1^. Cell compartmentalization of organelles itself has been proposed as a mechanism to restrict the opportunities of cheating mutants^1^. Fungi are excellent model systems to study the importance of these different factors, as they vary in the extent of cell compartmentalization. Furthermore, the dual nature of multicellular development, both by clonal outgrowth and by fusion among individuals, facilitates precise manipulation of relatedness, as our experiments demonstrate.

## Methods

### Experimental evolution

To test whether the fungus *N. crassa* is vulnerable to the evolution of somatic cheater morphotypes we performed an evolution experiment, in which a culture was regularly transferred to fresh growth medium to allow selection of new mutations. To test whether relatedness among the nuclei of a single individual affects the evolution of cheating we compared selective conditions with high and low relatedness. Both selective conditions were similar in growth conditions such as medium, temperature and growth time. Importantly, nuclei went through a single-celled bottleneck as asexual spores at each transfer. These spores were inoculated evenly on solid medium at a high density (∼2.85 × 10^5^ per cm^2^) with the result that during or soon after germination, germlings or young colonies were in contact with each other (Fig. 1). The key difference between the two selective conditions was the ability of colonies to fuse with each other. Fusion counteracts increased relatedness within colonies after the single-celled bottleneck (Fig. 1).

For the high-relatedness condition, we used a mutant strain, the hyphae of which lacked the ability to fuse. Each spore of this mutant formed a separate colony with clonally related nuclei, with its own reproductive output (Fig. 1). Competition therefore mainly occurred between colonies, favouring genotypes with a high spore yield (Fig. 1). This condition mimics a natural situation in which clonal outgrowth and limited fusion between colonies resulting from allorecognition maintain high-relatedness among the nuclei of a single individual.

For the low-relatedness condition, we used a regular laboratory strain, the hyphae of which are able to fuse. Since we used a single strain, all spores had the same allotype so that all germlings could fuse with surrounding germlings, resulting in a single coherent colony, shared by the nuclei of all germlings. Since there are no physical borders dividing different fungal individuals anymore, competition now primarily occurred at the level of nuclei, competing for the reproductive output of the shared individual, and less at the level of individuals (Fig. 1). Initially, the nuclei were highly related (that is, genetically similar), as they all descended from a single-spore culture, but mutation will soon supply genetic variation lowering relatedness among nuclei. New mutations could lead to a somatic cheater morphotype, defined as a variant that has a selective advantage within the mycelium by using somatic resources for its own replication while contributing less to somatic functions (Fig. 1).

Strains were obtained from the Fungal Genetics Stock Center (FGSC) and strain numbers refer to the FGSC catalogue^30^. For the high-relatedness treatment we used the fusion mutant *soft* (FGSC11293) (ref. 31). For the low-relatedness treatment we selected a strain from a cross between a standard lab strain (FGSC2489) and a strain with multiple markers in the background of the standard lab strain (FGSC5130). The selected strain contained a mutation that induces inositol dependence for growth, which we used as a selective auxotrophic marker. For both treatments, a culture that originated from a single spore was made to start the evolution lines, and asexual spores from these cultures were stored at −80 °C in a glycerol/pepton solution: 25% glycerol, and 7% Bacto neopepton (BD, Sparks, MD 21152 USA). For both treatments, we started eight parallel evolving cultures from this single-spore culture.

The growth medium used for experimental evolution was Vogels Minimal Medium (VMM) with 2% sucrose as carbon source and solidified with 2% agar^32^. VMM contains all the necessary ingredients for optimal growth during the asexual lifecycle. Cultures were grown in glass tubes (150 × 17.5 mm) with 7.5 ml of VMM medium solidified under an angle of ∼60° (slanted culture tubes), resulting in ∼7 cm^2^ of surface area. Evolution lines were maintained by serially transferring 1% of the produced asexual spores with growth phases in between transfers. Asexual spores were inoculated by transferring 50 μl suspension with a micropipette and spread out this suspension over the surface with the same pipette tip. The first inoculation was done with a spore suspension made from a culture grown from asexual spores from the −80 °C stock. To a 4-day-old culture, 5 ml of sterile water was added and the tube was vortex mixed for ∼15 s. The cultures were incubated for 3 or 4 days at 25 °C with cycles of 12 h light and 12 h darkness. After this, asexual spores were collected from the cultures in 5 ml of sterile water by vortex mixing ∼15 s. This yields roughly 4 × 10^7^ asexual spores per ml. Fifty μl of these spore suspensions was then used to inoculate fresh culture tubes similar as the previous ones, resulting in a density of ∼2.85 × 10^5^ asexual spores per cm^2^. From the same suspensions 800 μl was frozen at −80 °C in a glycerol/pepton solution. This serial transfer was then done twice per week alternating 3 and 4 days of growth in between transfers. Initially 31 transfers were done for all 16 evolution lines. If a simple binary fission model for division is assumed and 1% of the spores grow to 100% of the spores each transfer, then log_2_100=6.64 generations or divisions occur between transfers. This equals 205 asexual generations after 31 transfers. This is a conservative estimate, since not all nuclei will end up in the spores and not all spores will go into suspension during spore collecting and not all spores have equal fitness. Later the lines were continued with asexual spores from the lines that were frozen at −80 °C after transfer 31, for ten more transfers up to a total of 41 transfers.

### Determination of spore yields

We determined the spore yield of all evolution lines to compare the effect on fitness between both treatments. The yield of asexual spores is an important fitness parameter for fungi^15^, which determines in our evolution lines how many spores are transferred to the next culture. To determine the yield we grew cultures in tubes under the same conditions as the evolution lines. For this, we spread spore suspensions from the samples frozen after transfer 31 on culture tubes. Spores from this culture were then used to make suspensions of 4 × 10^7^ asexual spores per ml of which 50 μl was spread on fresh slanted culture tubes. After 4 days of growth, asexual spores were collected by adding 5 ml of sterile water to the culture tube followed by ∼15 s of vortex mixing. This suspension was then used to estimate spore yields using a haemocytometer. Per line, the yield was determined for three replicate cultures.

### Isolation of morphotypes

We analysed colonies grown from single spores for morphological differences to find out whether the evolved cultures consist of more than one morphological distinct type and isolated these different morphotypes. This was done by spreading diluted spore suspensions obtained from the evolved cultures after transfer 31 on counting plates. Counting plates are Petri dishes with VMM adjusted, by replacing sucrose with sorbose and adding 0.05% fructose and 0.05% glucose (sorbose-VMM) on which growth is restricted, so that single-spore colonies remain small^32^. These plates were incubated for 1 week at 25 °C with cycles of 12 h light and 12 h darkness to allow for full development of the phenotype. For each line several plates were made so that more than hundred colonies could be analysed for their morphology (see Fig. 2b for an example). From each distinguishable morphotype (by eye or dissecting microscope) on these plates several colonies were picked up to grow in standard slanted culture tubes that were described before. These were grown for 4 days at 25 °C with cycles of 12 h light and 12 h darkness after which cultures grown from similar morphotypes on plate were compared with make sure that one morphotype did not have hidden differences that would be visible in the environment they were selected in. Diluted suspensions from these tube cultures were spread on counting plates again to make sure it was a pure culture and to confirm that the phenotype was stable.

### Testing linear growth rate and yield of the morphotypes

We tested the obtained morphotypes for yield of asexual spores to compare them with the evolved cultures they were part of. In addition, we tested the linear growth rate to see if this trait had changed as well in the evolved types. Yield was determined in the same way as described above for the yield of the evolved cultures. Linear growth rate is defined as the distance a fungal culture can grow in one direction per time unit. To determine this we grew the morphotypes in so-called race tubes that contain long stretches of agar. The race tube was made from a disposable 50 ml pipette in which 20 ml of VMM medium is solidified over the length of the pipette by laying the pipette horizontally on the bench during solidification^33^. The different morphotypes were inoculated in race tubes using an inoculum of ∼2 × 10^5^ asexual spores suspended in 20 μl of water. Growth in three replicate tubes was measured daily for 3 days.

### Testing competitive success relative to the ancestral strain

We tested the morphotypes from the evolved low-relatedness cultures for their competitive success relative to an ancestral strain in direct competition. The ancestral competitor was selected from progeny from a cross between lab strain FGSC2489 and multiple marker strain FGSC5130. The strain is isogenic to the strain used in the evolution experiment except for a different selective marker, which is a pantothenic acid requirement. The competing strain is isogenic and thus of the same allotype, so that the evolved strain can fuse to its competitor similarly as during the evolution experiment. Competitive success was measured in a setting identical to the selective environment of the evolution experiment in a ratio of 1:9 (evolved morphotype:ancestral). This low frequency of the evolved morphotype was chosen to maximize potential benefits from exploiting the competitor, and to mimic the early selective phase. Competitions were set up between the evolved morphotypes and the competitor and, to correct for marker effects in the analysis, also between the ancestor of the evolution lines and the competitor with the other marker. Competitions were set up in slanted culture tubes with VMM medium. Asexual spores from the morphotype and the ancestral strain were mixed in a ratio of 1:9 in a suspension of 4 × 10^7^ asexual spores per ml, of which 50 μl was used to spread on a slanted culture tube. The competition culture was then grown for 4 days at 25 °C with cycles of 12 h light and 12 h darkness. Asexual spores were suspended in 5 ml of water. To determine the numbers of each competitor before and after the competition, an appropriate dilution of this suspension was inoculated on five counting plates with sorbose-VMM supplemented with pantothenic acid and five counting plates supplemented with inositol, allowing growth of only the ancestral competitor and only the evolved morphotype, respectively.

In addition, to test if differences in competitive success depended on fusion with the tested strain, we randomly selected a few strains to compete with a strain they could not fuse with. Two typical cheat morphotype 10.2 and 13.2 and one social morphotype 10.1 were chosen to test the competitive success in competition with a strain of a different allotype similarly as the first series of competitions. The competing strain is a strain with the same genetic background as the strain used in the evolution experiment except for differences at three allorecognition genes and a pantothenic acid requirement marker (FGSC1425) (ref. 34).

Competitive success of a strain was calculated as the ratio of that strain to its competitor after competition divided by that ratio before competition, using the following equation:


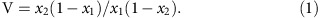


Where *x*_1_ is the frequency of the competing strain before competition and *x*_2_ its frequency after competition. Competitive success was normalized to the success of the ancestral strain used for the evolution experiment.

### Determining the frequency of cheater in evolution lines

We determined the frequency of the typical cheater morphotype (morphotypes that show a significant reduction in yield when grown in monoculture) for all the lines that contained cheater morphotypes after transfer 31 and after transfer 41. For lines 10, 12 and 13 we determined the cheater morphotype frequency at multiple time points in the evolution experiment to see how their frequency had changed over time. Frequencies were determined by plating appropriate dilutions of spore suspensions obtained from the −80 °C samples made after each transfer on counting plates. These plates were incubated for 1 week at 25 °C with cycles of 12 h light and 12 h darkness to allow for full development of the phenotype. If more than one cheating morphotype existed in an evolved line, these were not easily distinguishable on the plate and thus were counted as one group.

### Determining frequency and density dependence of competitive success

To test whether the competitive success of a cheater morphotype, relative to a social morphotype is dependent on frequency and/or density we set up competitions directly between the cheater morphotype and the social morphotype originating from the same evolution line under various inoculation densities and various starting frequencies of the cheater morphotype. This was done with the morphotypes 10.1 and 10.2 from line 10 and morphotypes 13.1 and 13.2 from line 13. The competitions were set up and analysed similar to the competitions with the ancestral strain described above, with the exception that the two competing strains now did not contain different markers. For this reason only one type of counting plate was used on which each type could be counted as the types had different morphologies. Competitions were set up under three different frequencies of the cheater morphotype (0.1, 0.5 and 0.9) and at three different inoculation densities (initial density, 3 × and 0.1 × in a full-factorial design. The frequency and density were manipulated as follows. Enough spores were collected from 1- or multiple 7-day-old cultures of the required types. For each type these were collected separately in suspensions of 1.2 × 10^8^ spores ml^−1^ water. Much more than this was not possible because dense spore suspensions are difficult to handle and the cheater morphotypes produce very small numbers of spores. From these suspensions three mixtures of the two types were made with the cheating type at frequency 0.1, 0.5 and 0.9. These were then diluted three times to the 4 × 10^7^ spores ml^−1^, which is similar to the concentration used in the previous competitions and represents the average starting density of cultures in the beginning of the evolution experiment. These diluted suspensions were then diluted ten times to 4 × 10^6^ spores ml^−1^. This resulted in nine suspensions representing all possible combinations of three cheat frequencies and three starting densities.

### Back crossings to ancestral type

All the typical cheater morphotypes were crossed with the standard lab strain to test whether the phenotype is caused by one or a few nuclear mutations. Crosses were set on slanted tubes with synthetic crossing medium^32^ with 2% of sucrose as a carbon source. The lab strain we used necessarily was of the opposite mating type as this is required for a successful cross. As *N. crassa* is hermaphroditical, we performed both reciprocal crosses, forcing the cheating type in the female role in one cross and in the male role in the other. For each cross the strain with the female role was inoculated by transferring some asexual spores to a fresh tube and then grown for about 1 week before it was fertilized with asexual spores of the strain in the male role. This fertilized culture was then grown for 1–2 weeks until mature fruiting bodies and sexual spores were visible.

Sexual spores were given the required heat shock at 60 °C for 30 min before an appropriate dilution was inoculated on two counting plates. These plates were incubated for 1 week at 25 °C with cycles of 12 h light and 12 h darkness to allow for full development of the phenotype.

## Additional information

**How to cite this article:** Bastiaans, E. *et al*. Experimental evolution reveals that high relatedness protects multicellular cooperation from cheaters. *Nat. Commun.* 7:11435 doi: 10.1038/ncomms11435 (2016).

## Figures and Tables

**Table 1:** Frequencies of typical cheater morphotypes in lines that have cheater morphotypes at two time points.

Line*	**Relatedness treatment**	**Frequency after transfer 31 (*****n***†**)**	**Frequency after transfer 41 (*****n*****)**
4	High	0.04 (312)	0.10 (523)
5	High	0.12 (342)	0.28 (627)
7	High	0.48 (287)	0.53 (475)
9	Low	0.87 (164)	0.36 (435)
10	Low	0.28 (163)	0.16 (409)
11‡	Low	0.32 (130)	0.21 (184)
12	Low	0.52 (136)	0.09 (500)
13‡	Low	0.63 (166)	1 (491)
14	Low	0.19 (144)	0.10 (484)
15	Low	0.16 (153)	0.05 (461)
16	Low	0.15 (126)	0.38 (295)

^*^Only lines that contain cheater morphotypes (types with significant lower yield compared with ancestor) are displayed in this table.

^†^*n* denotes the number of single-spore cultures examined.

^‡^These lines contain >1 typical cheater morphotype, their combined frequency is displayed because they were not distinguishable from each other on a counting plate.
